# Supplementary material for: Oncogenic Impact of TONSL, a Homologous Recombination Repair Protein at the Replication Fork, in Cancer Stem Cells
Source: Int J Mol Sci. 2023 May 31;24(11):9530. doi: 10.3390/ijms24119530 (PMC10253825; doi:10.3390/ijms24119530)
Supplement: Supplementary file 1 [file ijms-24-09530-s001.zip › ijms-2321182-supplementary.pdf]

Supplementary Figure S1

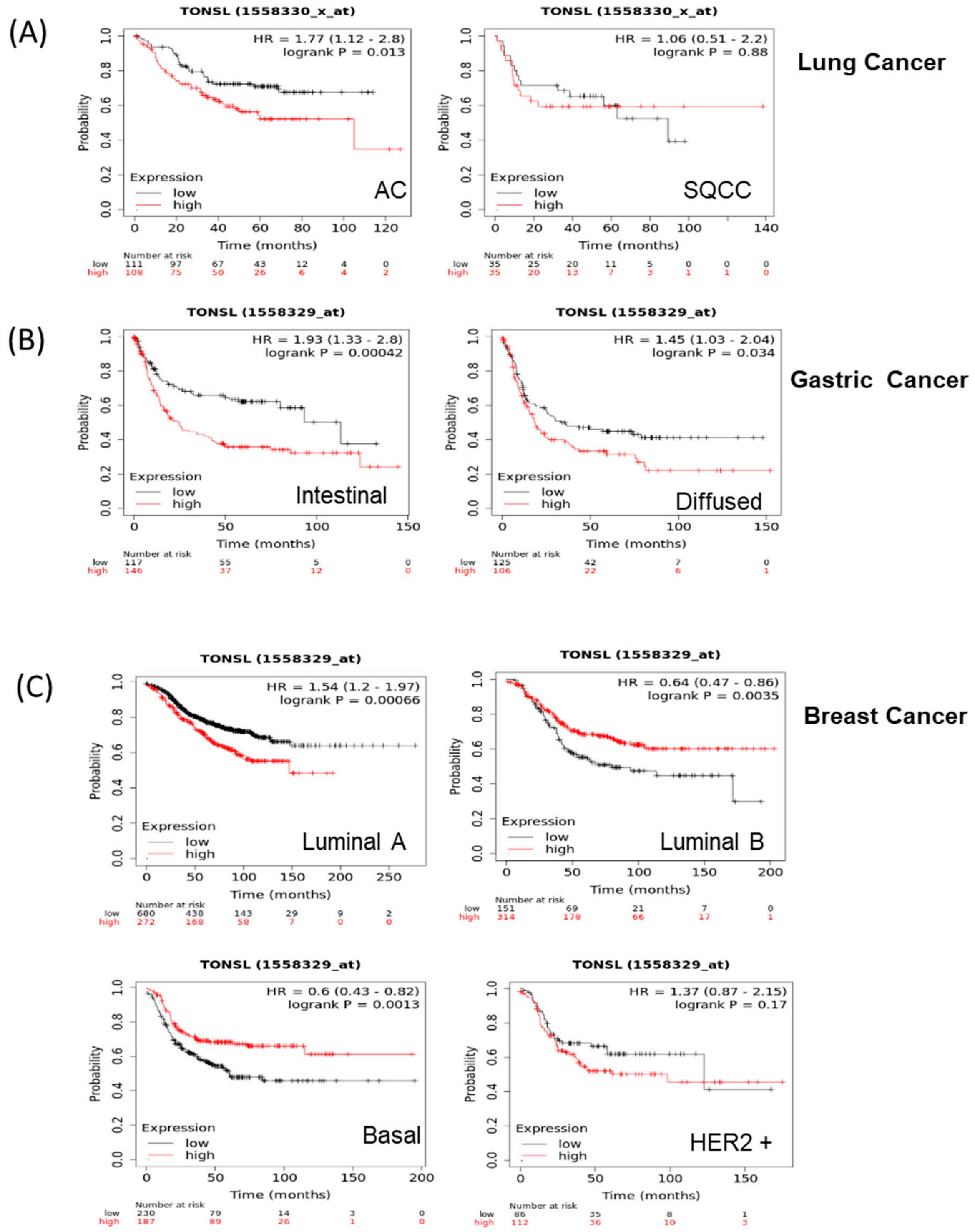

Figure S1. TONSL mRNA expression (from gene chip) was associated with cancer prognosis, recurrence free survivals. Kaplan-Meier survival curves of TONSL mRNA expression levels for (A) lung cancer (AC, *n*=219 ; SQCC, *n*=70), (B) gastric cancer (intestinal, *n*=263 ; diffused, *n*=231), (C) breast cancer subtypes (luminal A, *n*=952; luminal B, *n*=465; HER2+, *n*=208; basal, *n*=417). Auto select best cut-off was used to split the cohorts of patients. All of the cohort showed a statistically significant difference except HER2+. (The log rank t test). (The data was accessed on 1 December 2021)

Supplementary Figure S2.

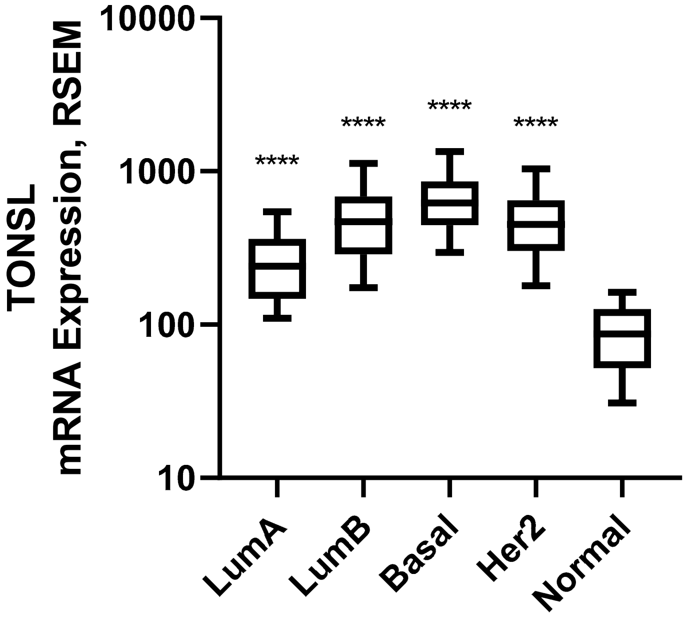

Figure S2. TONSL mRNA expression in various breast cancer subtypes and normal tissue. TONSL mRNA expression in breast cancer and normal breast tissues is shown as boxplots. The whiskers are 10-90 percentile range. RSEM (Batch normalized from Illumina HiSeq_RNASeqV2) RNA seq data was derived from TCGA PanCancer Atlas study (The data was accessed on 16 December 2022). For statistical analysis, Dunnett's multiple comparisons test is used. LumA (n=499), LumB (n=197), Basal (n=171), Her2 (n=78), Normal (n=112). ****, P<0.0001.

Supplementary Figure S3.

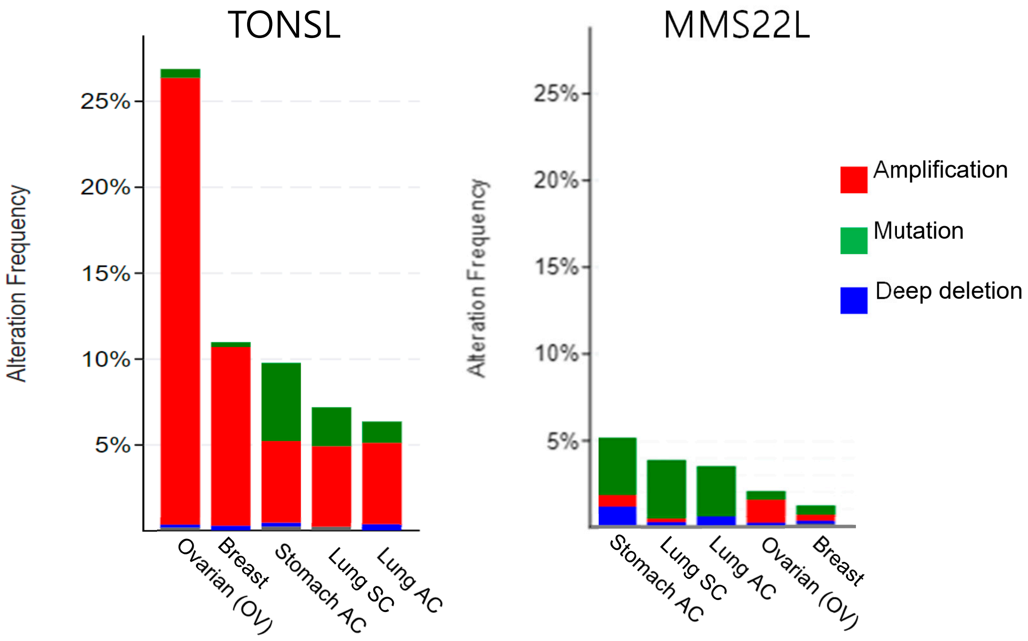

Figure S3. Gene amplification and gene amplification, deletion and mutation of *TONSL* and *MMS22L*. The mutation and alteration frequency were acquired from TCGA Pan-cancer Atlas Study using cBioportal. SC, Squamous Carcinoma; AC, Adenocarcinoma; OV, Ovarian Cancer. The frequency represents the percentage to the total tumors tested.

Supplementary Figure S4

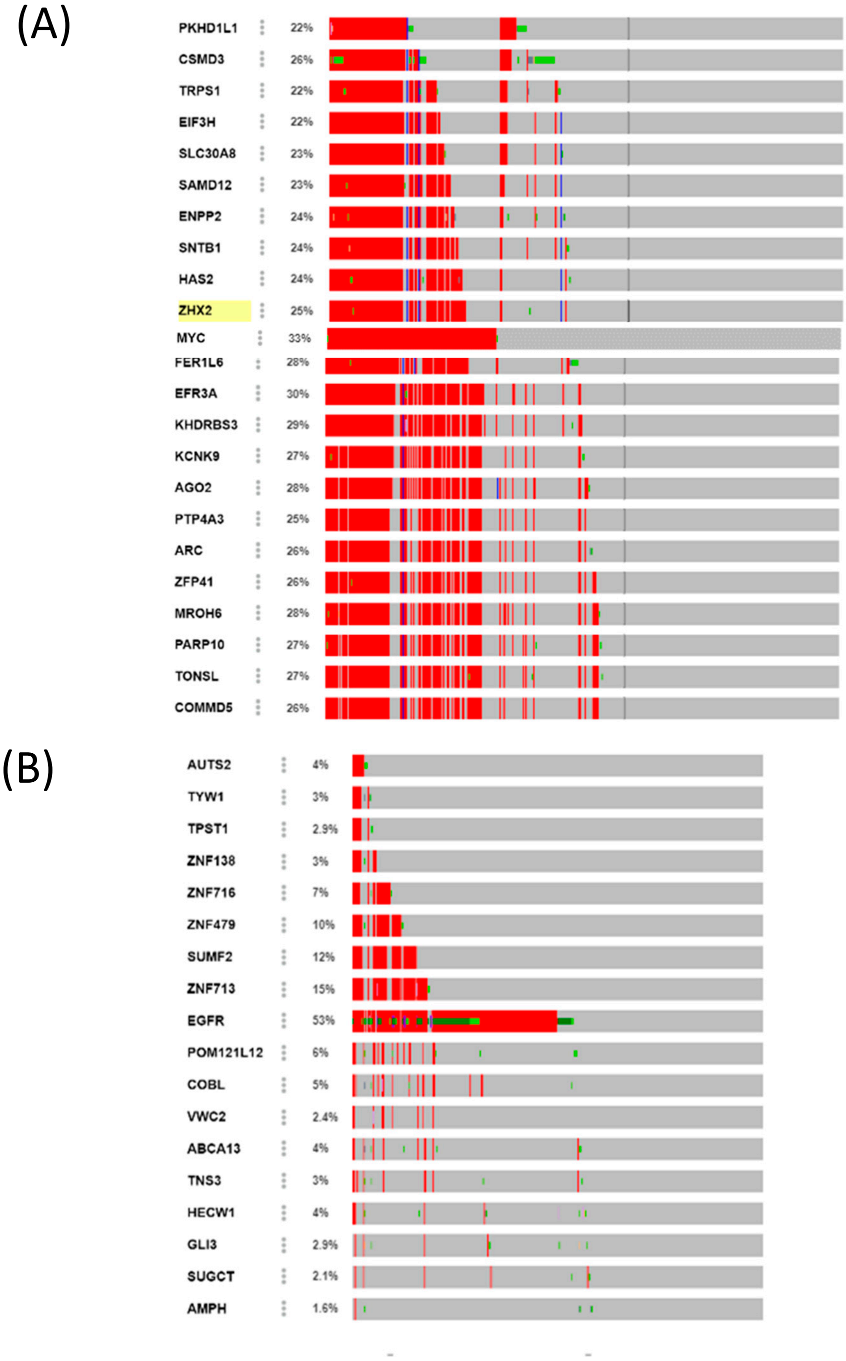

Figure S4. Gene coamplification pattern with neighboring potent driver oncogenes (A) genes close to the MY C in Ch8 in ovarian cancer. (B) Genes close to EGFR in Ch7in glioblastoma. The data is from TCGA Pan-cancer Atlas Study and the analyzed data was captured from cBioportal.

Supplementary Figure S5

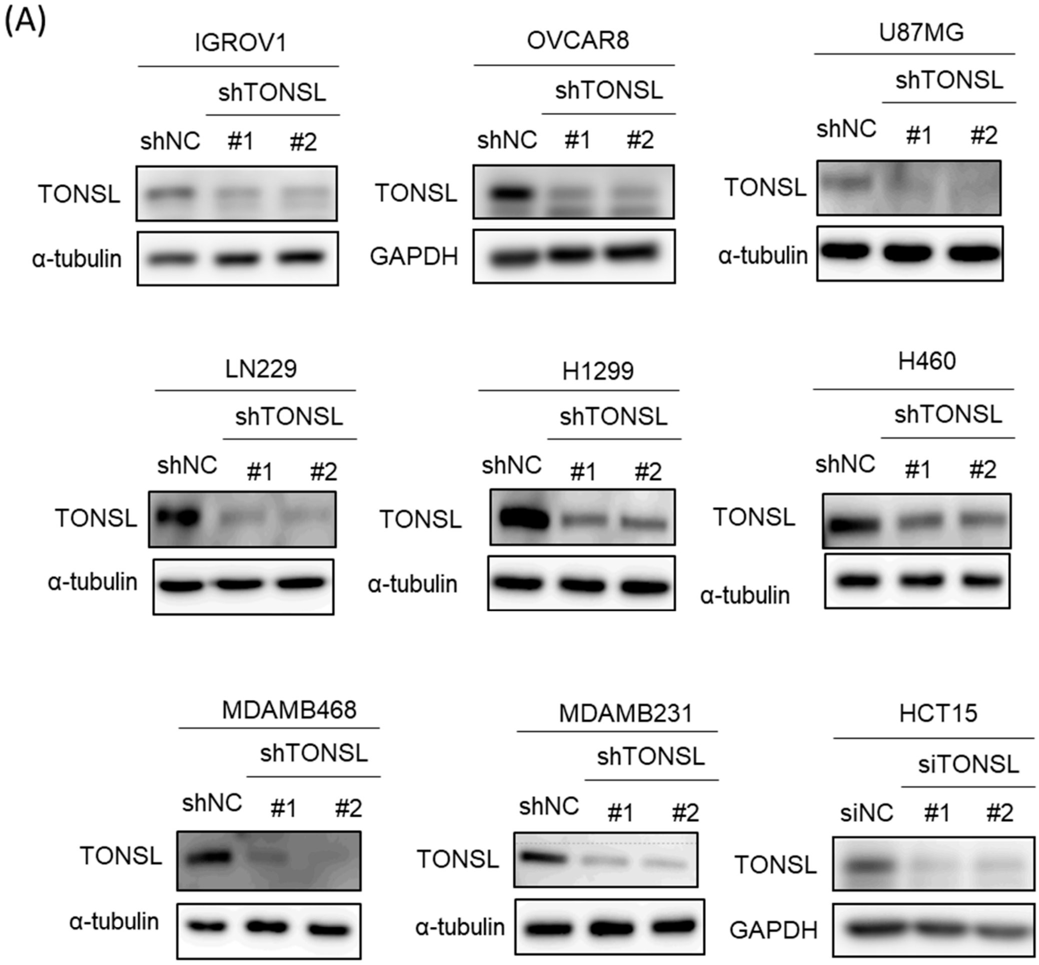

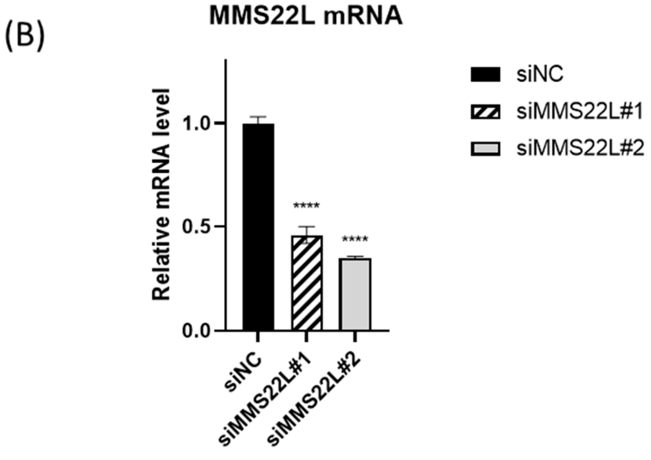

Figure S5. The effect of siRNA and shRNS used in the study (A) TONSL western blot (B) MMS22L real-time RT-PCR

Supplementary Table S1

Table S1. The distance and co-amplification frequency with *MYC* at 8q24. The data is from TCGA Pan-cancer Atlas Study and the analyzed data was captured from cBioportal.

GENE	Distance from MYC (Kb)	Co-amplification with *MYC* (%)
ANGPT1	-20479.5	54.41504
EMC2	-19286.9	54.41504
PKHD1L1	-18240	61.0108
CSMD3	-15515.9	65.95762
TRPS1	-12330.6	70.90444
EIF3H	-11240	73.37785
SLC30A8	-10602.2	75.0268
SAMD12	-9360.47	77.50021
ENPP2	-8200	79.14915
SNTB1	-7140	80.79809
HAS2	-6140	84.09597
ZHX2	-4978.24	82.44703
FER1L6	-3777.01	84.92044
MTSS1	-3168.15	88.21832
MYC	**0**	**100**
EFR3A	3192.55	93.98961
KHDRBS3	7819.329	89.04279
KCNK9	11022.13	86.56938
AGO2	12824.41	75.37519
PTP4A3	13672.79	79.97362
ARC	14870.85	78.32468
PARP10	16251.94	76.67574
TONSL	16697.25	75.85127
COMMD5	17108.36	75.85127

Supplementary Table S2

Table S2. Sequences of quantitative RT-PCR primers and siRNAs

Gene	Forward primer (5’-3’)	Reverse primer (5’-3’)
MMS22L	atgtgagcgggaatctctt	gaaacacttggggttcgtc
β-Actin	attggcaatgagcggttc	ggatgccacaggactccat

Target gene	siRNA	Sense (5’>3’)	Antisense (5’>3’)
TONSL	siTONSL#1	caacuacgggcaucuaga	gacaauuucuagaugccc
	siTONSL#2	uuacuucaggaagagcau	aaggaagaugcucuuccu
MMS22L	siMMS22L#1	ucacaaaguccuuggaauauu	uauuccaaggacuuugugauu
	siMMS22L#2	gggagaaagcaaaggaauuu	auuccuuugcuuucucccuu
